# A Novel Computer-Aided Approach for Parametric Investigation of Custom Design of Fracture Fixation Plates

**DOI:** 10.1155/2017/7372496

**Published:** 2017-01-19

**Authors:** Xiaozhong Chen, Kunjin He, Zhengming Chen

**Affiliations:** ^1^College of Internet of Things Engineering, Hohai University, Changzhou, China; ^2^School of Intelligent Equipment and Information Engineering, Changzhou Vocational Institute of Engineering, Jiangsu, China; ^3^Changzhou City Key Lab of Orthopedic Implants Digital Technology, Changzhou, China

## Abstract

The present study proposes an integrated computer-aided approach combining femur surface modeling, fracture evidence recover plate creation, and plate modification in order to conduct a parametric investigation of the design of custom plate for a specific patient. The study allows for improving the design efficiency of specific plates on the patients' femur parameters and the fracture information. Furthermore, the present approach will lead to exploration of plate modification and optimization. The three-dimensional (3D) surface model of a detailed femur and the corresponding fixation plate were represented with high-level feature parameters, and the shape of the specific plate was recursively modified in order to obtain the optimal plate for a specific patient. The proposed approach was tested and verified on a case study, and it could be helpful for orthopedic surgeons to design and modify the plate in order to fit the specific femur anatomy and the fracture information.

## 1. Introduction

Distal femur fractures consist of around 3% of all femur fractures and are results of the high-energy trauma to young patients and the low-energy trauma to older population with osteoporosis bones [1, 2]. Although internal fixation technologies are used in orthopedic trauma surgery, these femoral fractures are difficult to treat for the comminuted fracture segments. In the past decades, the development of distal femur implants has facilitated the operative treatment of such fractures [3]. Compared to other implants such as intramedullary devices, plates are more widely applied to clinical treatments for some reasons [4]. However, in some practical cases, additional remodeling was frequently taken to proximate plates to fit the specific anatomy and the patient's fracture information. Nevertheless, these modifications will lead to the increased failure rate of surgical treatments [5]. Plates precontoured based on specific femurs may promote the anatomic recovery and achieve fracture healing [6]. Therefore, the development of patient-specific plates has been regarded as a research trend of next-generation medical devices [7].

Some investigations [8–10] have been focused upon the plate development recently. In the work [9], the 3D solid model of femoral fracture reconstructed from Computed Tomography (CT) scan data was utilized to create the plate. Shen and coworkers [10] developed dissection plates for different patients based on CT slices. In above two studies, theoretical reference and operation reference models were proposed for the orthopedic surgery and fracture fixation assembling. However, little attention has been devoted to consider the efficiency of plate development. In recent published studies, the custom plate design was achieved with various processes, that is, reduction of fractured bones, creation of fixation plates, and modification and fracture plates [11]. However, most researchers have neglected to find the relationship between various processes to improve design efficiency owning to different discrete procedures in the overall development. Thus, how to construct and edit plate models accurately and conveniently is very significant, and it is a challenge to the development of a specific patient plate.

Previously, the authors proposed a parametric method to construct a surface model of a patient's femur based on surface feature parameterization [12]. It was found that the surface model could be accurately constructed and modified according to the hierarchical feature parameters of a patient's femur [13]. In this study, a novel parametric method is proposed to develop a patient-specific plate based on the patient's information by using feature parameterization. The parametric models of femur plates can be quickly created based on parameter maps between the femur and the plate, modified with a few semantic parameters, and optimized by recursively modifying plate shapes based on feature parameters. To achieve this purpose, three major requirements have to be satisfied:

- The plate should be designed according to the femur anatomy and fracture information (such as fracture type and degree) of specific patients.
- The plate should be edited and modified conveniently by the physicians to match the morphology of a specific patient bone.
- The creation and modification of the plate have to be done within 24 hours after the injury to void complications.

Although there are lots of meaningful data in any fractured femur models, these data are hard to measure and not defined as parameters, so that they are difficult to be used in the representation and design of plates. The research work reported in this paper is motivated by the above observation. Combining with the thought of feature parameterization [14], the main contribution of this paper lies in the development of a rapid method for the plate design from the fractured femur of a specific patient. Our proposed method can support the design user to customize and optimize femur plates according to the information of specific patient.

## 2. Materials and Methods

In this study, an intact right femur of a young female volunteer without any disease or trauma, was selected at our research center.

**Figure 1:**
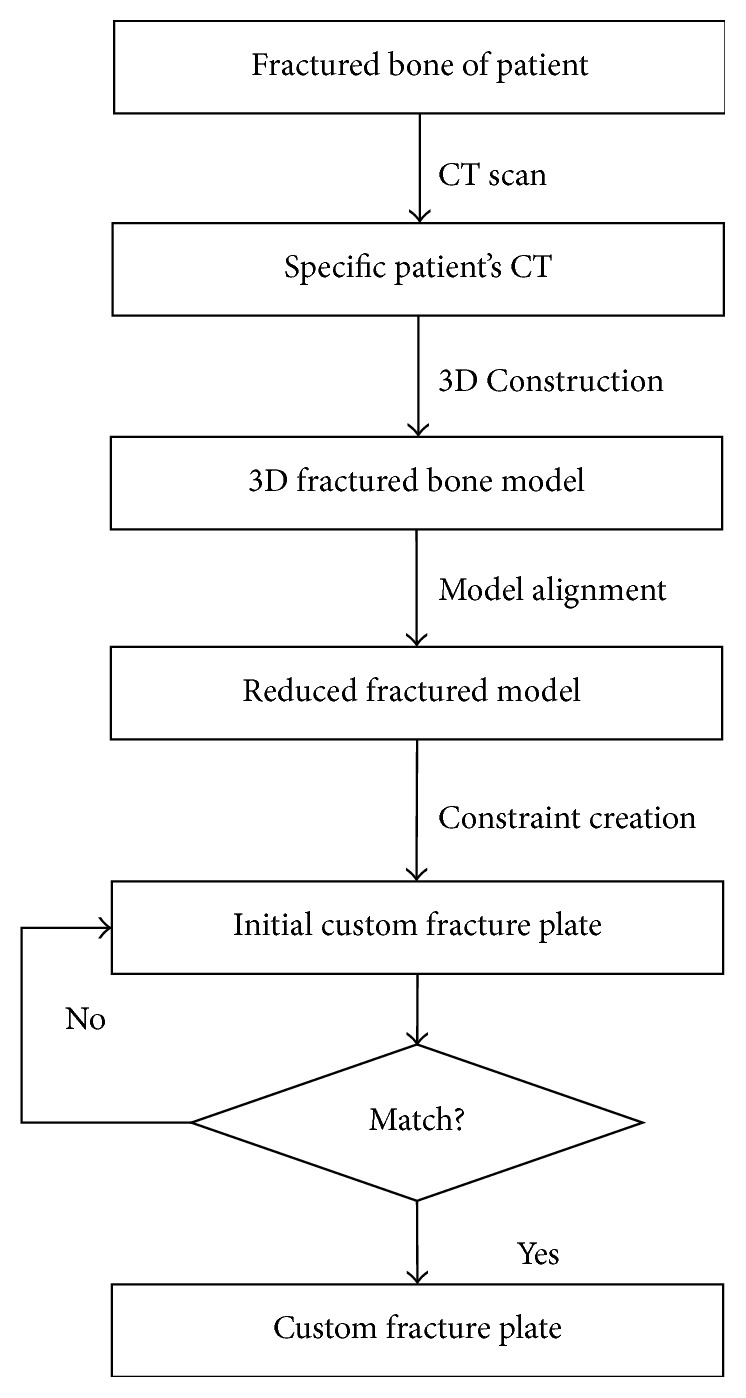
The flowchart of the design of a custom fracture plate.

The complete framework for designing the custom plate based on the existing fractured femur of a specific patient is described in Figure 1. The major steps will be explained further in the following parts of this section.

### 2.1. Construction of the Fractured Bone Model

To obtain 2D scan images of the femur, 64-slice CT (MSCT, Aquilion 64, Toshiba, Zoetermeer, Netherlands) was utilized. After CT scanning of the selected femur, by using the medical processing software MIMICS, the intact 3D femur mesh model was created with four stages, such as image preprocessing, image segmentation, surface extraction, and mesh simplification [15].

**Figure 2:**
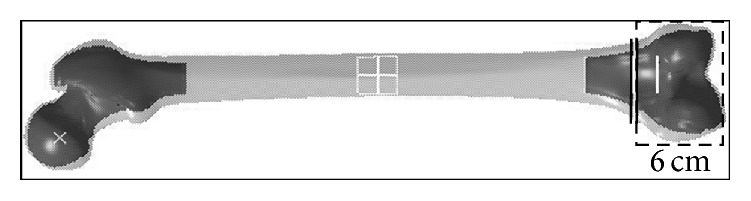
The simulated 3D fractured femur mesh model with a 33A1-C3 type fracture.

A fracture gap was created 6 cm above the knee joint, and the fractured segments were translated and rotated to simulate a 33A1-C3 type fracture (AO-Classification) as shown in Figure 2.

### 2.2. Reduction of Fractured Bone Segments

In many femoral fractures, the segments are usually comminuted and dislocated, and the 3D fractured model cannot describe the accurate morphologies of a normal femur. Therefore, the 3D reconstruction of the intact femur representing the normal surface shape of a specific patient, especially of the fractured site, is very useful for surgeons to select an optimal implant and treat the fracture.

With the consideration of the significance of femur shapes in the plate design, the authors focused on the surface model of a intact femur, and the surface model with the fracture evidences was constructed with the following step.

**Step 1 .**

**Figure 3:**
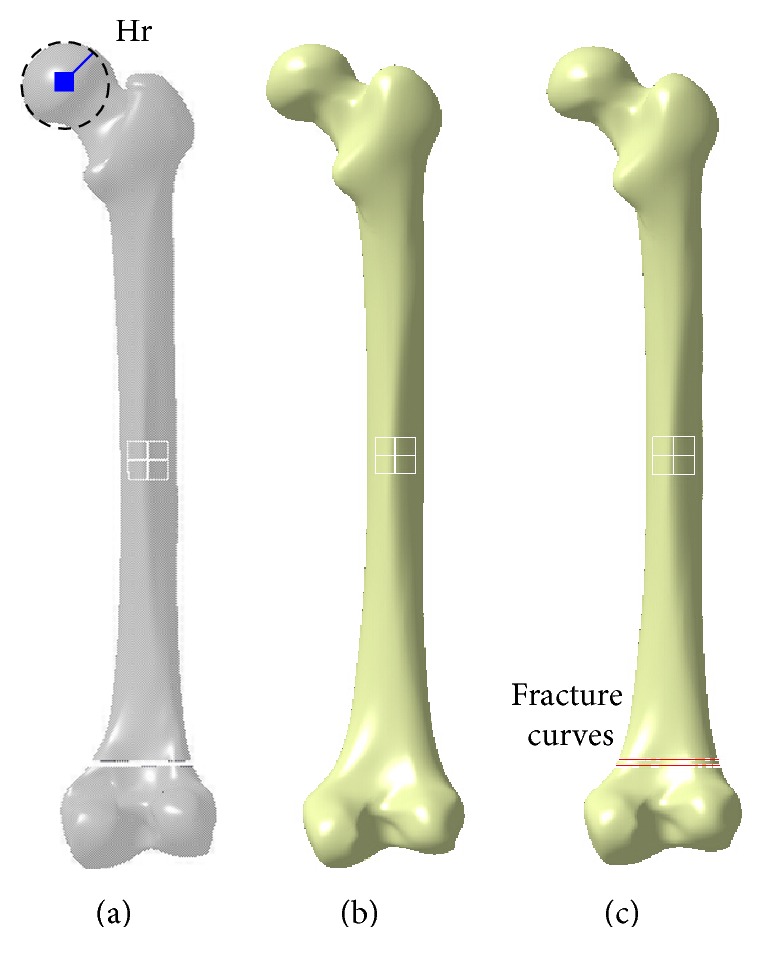
The detailed steps of constructing the surface model of restored fractured femur. (a) Parameters measurement, (b) surface mode construction, and (c) fracture evidence restoration.

A few of valid femur parameters, were extracted from the fractured solid femur constructed in Section 2.1, as shown in Figure 3(a).

**Step 2 .**

The surface model of the fractured femur was constructed according to the patient's femur parameters based on the parameter constraints to represent the normal shape of the femur as shown in Figure 3(b).

**Step 3 .**

The constructed surface model was validated and modified based on femur parameters.

**Step 4 .**

The fractured femur model with fracture curves which were used to represent major fracture evidences as shown in Figure 3(c) was extracted by projecting the main boundary curves onto the surface model.

### 2.3. Construction of the Initial Plate Model

**Figure 4:**
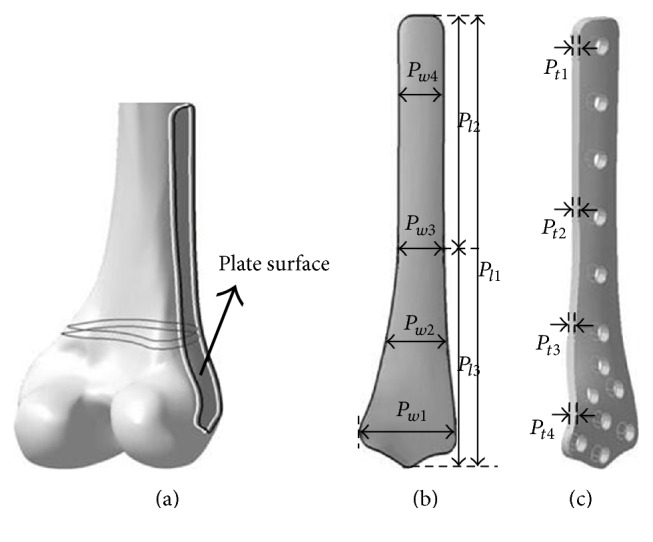
Parameterization of the distal femur plate for a specific fracture. (a) Contour projection and surface generation; (b) parameterization of the plate surface; (c) parameterization of the volume model.

Because the constructed femur model in the above subsection may describe anatomic shapes of the detailed femur and the main information of the fracture, the plate bottom surface between the bone and the femur was selected with a suitable contour based on the fracture from the above surface model, and it was extracted with various distances, so that the plate volume model was constructed. Finally, the feature parameters were defined to represent the plate model as described in Figure 4. The algorithm for constructing and parameterizing the plate was expounded as follows.

**Step 1 .**

A closed curve on the lateral surface was created from the orthographic projection of the contour sketch, and the surface of the femur was cut with the projection and cut operation to obtain the plate bottom surface as shown in Figure 4(a).

**Step 2 .**

The bottom surface of the plate was parameterization to represent the detailed shape with semantic parameters, such as length parameters and width parameters as shown in Figure 4(b).

**Step 3 .**

The bottom surface was extruded outwards with nonisometric distances to obtain a volume model of the plate, and volume features (that is thickness) of the model were presented with semantic parameters as shown in Figure 4(c) to create the model of the specific plate.

**Step 4 .**

**Figure 5:**
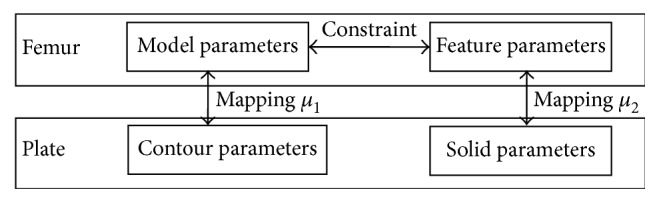
Parameter map relations between the femur and plate. *μ*_1_ is a map between the model and contour parameters, whereas *μ*_2_ is a map between the femur feature and solid parameters.

**Figure 6:**
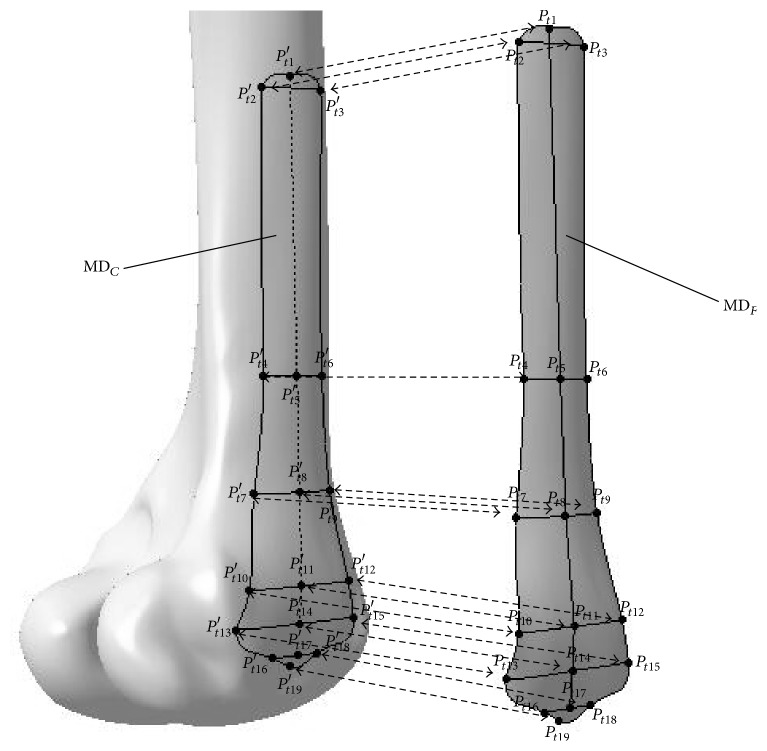
Map relations between the feature parameters of the femur surface and the plate surface.

**Figure 7:**
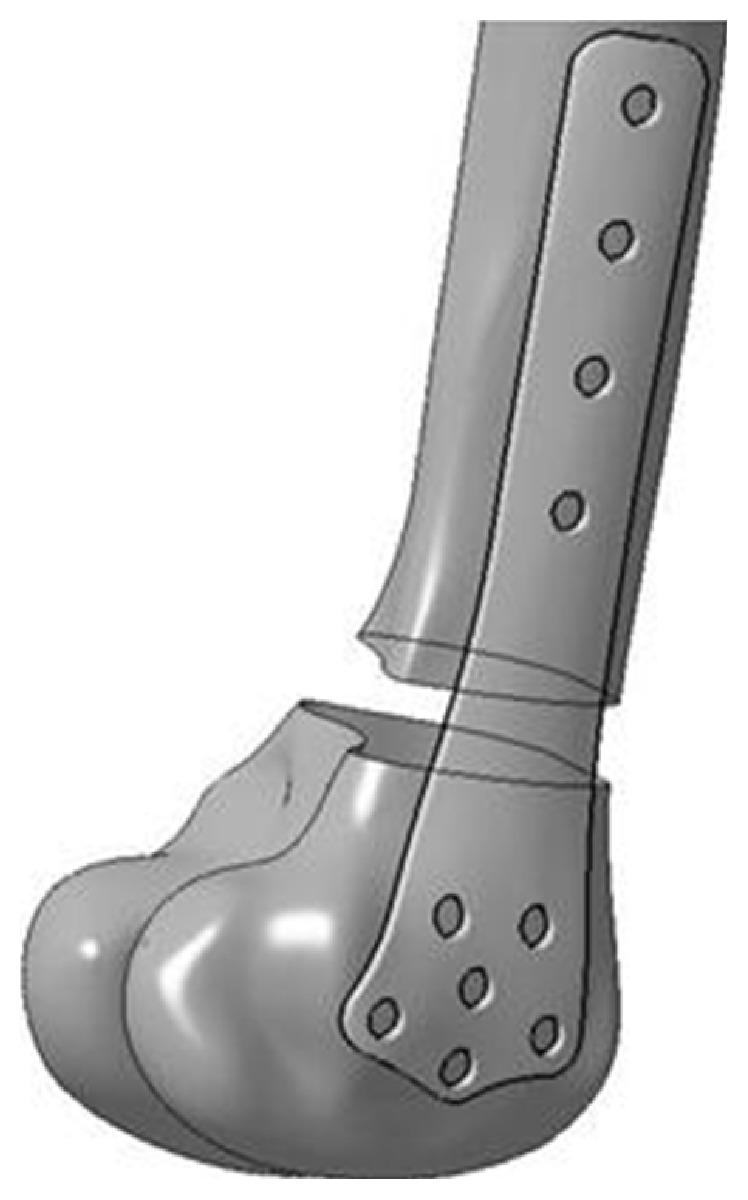
The specific plate model atomically constructed based on the parameter maps and femur parameters.

Based on the approximate linear map relations between femur parameters and plate parameters proposed in our previous study [11] as described in Figure 5, the detailed parameter values can be atomically evaluated. According to the map relationship between feature points of the femur surface and the plate surface as shown in Figure 6, the shape and the thickness of specific plate were adjusted to match the femur anatomy of a specific patient and the fracture information as shown in Figure 7.

In this study, the femur model with a 33A1-C3 type fracture was firstly measured to obtain valid feature parameters of femur, that is, the head radius Hr (21.061 mm), and then the SFMF was quickly constructed for the plate design [12]. Based on the map relations between femur parameters and plate parameters, the specific plate was constructed to match the femur anatomy of a specific patient, and the detailed parameter values were listed in Table 1.

### 2.4. Shape Modification of the Plate

**Figure 8:**
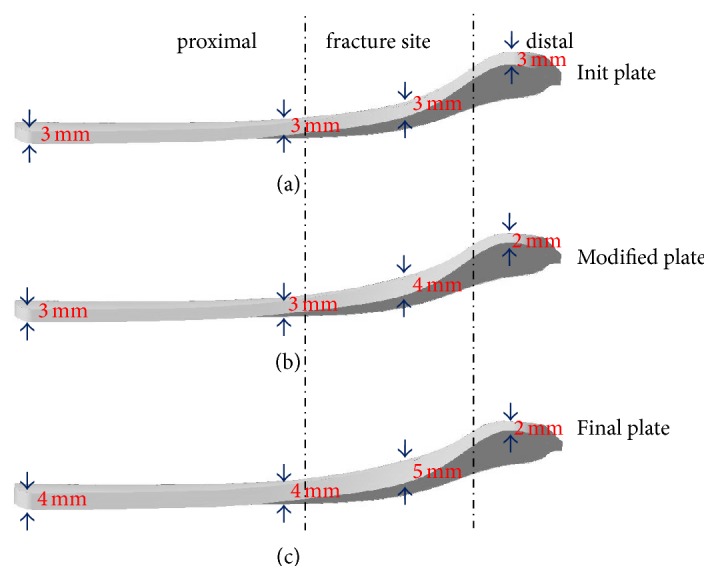
Three instances with various values of thickness parameter. (a), (b), and (c) represent the init, modified, and final plate, respectively.

The plate was divided into three parts, for example, the proximal, fractured site, and the distal. Based on the feature parameters of the plate, the initial plate atomically created with the femur parameters can be efficiently edited and modified. As shown in Figure 8, the thickness values of each part were ranged from 3 to 4 mm, 3 to 5 mm, and 2 to 3 mm, respectively.

In the first instance, that is, the init plate constructed in Section 2.3, 3 mm was assigned to all parameters in various parts, as shown in Figure 8(a). In the second instance, the thickness of the distal was reduced from 3 to 2 mm because the attached area of that part was larger than any one of the others, as shown in Figure 8(b), while the width value of the fractured site was increased from 17 mm to 21.8 mm to improve the strength of the plate. In the third instance, that is, the final plate, the parameter values of the fractured site were increased as shown in Figure 8(c).

The init and final instances of the distal plate with each width parameter shown in Table 2 were used to evaluate the mechanical stress in the fractured model fixated with plates and screws. The maximum von Mises' stresses for the same fractured model were obtained. The parameterization modification resulted in a remarkable reduction of maximum von Mises' stress from the init to the final, so that the fatigue life of the final plate can be improved.

In this study, the init plate exhibited significantly lower maximum von Mises' stress than the that reported in the previous work [16], and the stress of the modified plate was significantly reduced compared to the one of the init plate.

What is more, the mechanical environment of the plate is related to various factors of specific patients, such as femur anatomy, bone quality, weight, fracture type, and complexity. The parameterization method can help nonexpert users to define and modify any values of the factors to effectively construct and optimize the plate according to the specific anatomy and fracture of patients.

## 3. Discussions

Designing plates that match the morphology of a specific patient bone is a great advancement in place of using commercially available plates which are designed based on average anthropometric data. Custom made total knees and total hips (joint arthroplasty, and most often in relation to the knee) are available and used frequently. But designing and producing these implants takes time to produce and manufacture. While the physician may have the time to wait for the design of custom plates if the case is elective (i.e., total knee or deficiency in the acetabulum), in the case of a fracture or fracture dislocation, the physician does not have time to wait. Most fracture cases should be done 24 to 48 hours after injury to avoid complications, especially long bone fractures.

In the published studies [8, 10], although the existing contours of commercial plates can be selected and developed as shape templates, the existing plate is hard to reuse for the freeform complexity of attached surfaces of plates. Therefore, it needs to start from scratch in a new development, even in case of the similar type of shapes for other patients with different sizes. Feature parameterization was an efficient way to represent and modify the complex shape of the plates. What is more, building up the linear maps between plate parameters and femur parameters can achieve the rapid development of origin plates based on a few femur parameters of specific patients.

The patient-specific plate which was designed in accordance with anatomical morphology and physiological radian on the femur, can well attach to the bone without additional shaping, as well as can be effective in maintaining fine relations between anatomic reduction of fracture fragments. However, it is a known fact that it is hard to accurately represent the anatomy shape of the fractured site, especially in commented fractures, because of the cancellous bone of the condyle. Fortunately, the SFMF created by statistical shape analysis can shield the differences and can describe the typical femoral anatomy of certain population. Therefore, its morphology can be used for developing and manufacturing of implants for a specific group [17].

In addition, to improve the mechanical strength of the plate, the holes near to the fracture gap should to be removed [5]. What is more, the fracture lengths result in the different stress of the fixation system [18]. Therefore the development of plates must match the fracture type and complex. Besides material, thickness is a very important factor in the plate design [16]; the thickness and the width parameters, especially in the fracture site, have significant meanings in the plate development. How to edit conveniently the shape of the plate is a challenge in the development of specified plates. By adjusting the values of the high-level parameters of origin plates, such as the width, thickness, and length, the final optimized plate can be constructed and modified effectively.

There are several limitations to the study as the results are obtained solely based on FEA. Firstly, the two constituents of the femur were assumed to be linear elastic and isotropic; however, the material of femur is very complex [19]. Secondly, the analysis was limited to axial loading case and torsional loading was not tested in this study; the main muscle load was not included [20]. Thirdly, the screw-bone interface was modeled as a bond constraint; although the constraint is a good approximation, it cannot represent the real mechanical environment of the fracture fixation [21]. Therefore, a future investigation could entail experimental testing of the plate to settle limitations.

## 4. Conclusions

The present study presents an integrated computer-aided approach to conduct a parametric investigation of the design of patient-specific plates. The study allows for improving the design efficiency of specific plates on the patients' femur parameters and the fracture information. Furthermore, the present approach will lead to exploration of plate modification and optimization. The three-dimensional (3D) surface model of a detailed femur and the corresponding fixation plate were represented with high-level feature parameters, and the shape of the specific plate was recursively modified based on finite element analysis to obtain the optimal plate for a specific patient. The proposed approach can be helpful for design users or surgeons to design and modify the plate rapidly and efficiently to match specific fractured femur bones.

## Figures and Tables

**Table 1:** The parameter values of the init plate for a specific femur anatomy (mm).

Parameters	Values
*P*_*l*1_	132.1
*P*_*l*2_	67.1
*P*_*l*3_	65.0
*P*_*w*1_	34.3
*P*_*w*2_	17.0
*P*_*w*3_	13.0
*P*_*w*4_	13.0
*P*_*h*1_	3.0
*P*_*h*2_	3.0
*P*_*h*3_	3.0
*P*_*h*4_	3.0

**Table 2:** The width values of two plates (the init and final) (mm).

Parameters	*P*_*w*1_	*P*_*w*2_	*P*_*w*3_	*P*_*w*4_
Init	13.0	13.0	17.0	34.2
Final	17.0	17.0	22.5	34.3
